# Relationship between insecticide resistance profiles in *Anopheles gambiae* sensu lato and agricultural practices in Côte d’Ivoire

**DOI:** 10.1186/s13071-023-05876-0

**Published:** 2023-08-09

**Authors:** France-Paraudie A. Kouadio, Nadja C. Wipf, Angele S. Nygble, Behi K. Fodjo, Christabelle G. Sadia, John Vontas, Konstantinos Mavridis, Pie Müller, Chouaïbou S. Mouhamadou

**Affiliations:** 1 Centre Suisse de Recherches Scientifiques en Côte d’Ivoire, 01 BP 1303 Abidjan 01 Abidjan, Côte d’Ivoire; 2 Université Nangui Abrogoua, Abidjan, Côte d’Ivoire; 3 Swiss Tropical and Public Health Institute, Kreuzstrasse 2, CH-4123 Allschwil, Switzerland; 4 University of Basel, Petersplatz 1, P.O. Box, CH-4001 Basel, Switzerland; 5 Institute of Molecular Biology and Biotechnology, Foundation for Research and Technology-Hellas, 70013 Heraklion, Greece; 6 Pesticide Science Laboratory, Department of Crop Science, Agricultural University of Athens, 11855 Athens, Greece

**Keywords:** Agriculture, Insecticide resistance, P450 genes, Gene expression, Target-site mutation, Malaria vectors, *Anopheles gambiae*, Côte d’Ivoire

## Abstract

**Background:**

Insecticide-based malaria vector control is increasingly undermined due to the development of insecticide resistance in mosquitoes. Insecticide resistance may partially be related to the use of pesticides in agriculture, while the level and mechanisms of resistance might differ between agricultural practices. The current study aimed to assess whether phenotypic insecticide resistance and associated molecular resistance mechanisms in *Anopheles gambiae* sensu lato differ between agricultural practices.

**Methods:**

We collected *An. gambiae* s.l. larvae in six sites with three different agricultural practices, including rice, vegetable and cocoa cultivation. We then exposed the emerging adult females to discriminating concentrations of bendiocarb (0.1%), deltamethrin (0.05%), DDT (4%) and malathion (5%) using the standard World Health Organization insecticide susceptibility test. To investigate underlying molecular mechanisms of resistance, we used multiplex TaqMan qPCR assays. We determined the frequency of target-site mutations, including *Vgsc*-L995F/S and *Vgsc*-N1570Y, and *Ace1*-G280S. In addition, we measured the expression levels of genes previously associated with insecticide resistance in *An. gambiae* s.l., including the cytochrome P450-dependent monooxygenases *CYP4G16*, *CYP6M2*, *CYP6P1*, *CYP6P3*, *CYP6P4*, *CYP6Z1* and *CYP9K1*, and the glutathione *S*-transferase *GSTe2*.

**Results:**

The *An. gambiae* s.l. populations from all six agricultural sites were resistant to bendiocarb, deltamethrin and DDT, while the populations from the two vegetable cultivation sites were additionally resistant to malathion. Most tested mosquitoes carried at least one mutant *Vgsc*-L995F allele that is associated with pyrethroid and DDT resistance. In the cocoa cultivation sites, we observed the highest 995F frequencies (80–87%), including a majority of homozygous mutants and several in co-occurrence with the *Vgsc*-N1570Y mutation. We detected the *Ace1* mutation most frequently in vegetable-growing sites (51–60%), at a moderate frequency in rice (20–22%) and rarely in cocoa-growing sites (3–4%). In contrast, *CYP6M2*, *CYP6P3*, *CYP6P4*, *CYP6Z1* and *CYP9K1*, previously associated with metabolic insecticide resistance, showed the highest expression levels in the populations from rice-growing sites compared to the susceptible Kisumu reference strain.

**Conclusion:**

In our study, we observed intriguing associations between the type of agricultural practices and certain insecticide resistance profiles in the malaria vector *An. gambiae* s.l. which might arise from the use of pesticides deployed for protecting crops.

**Graphical Abstract:**

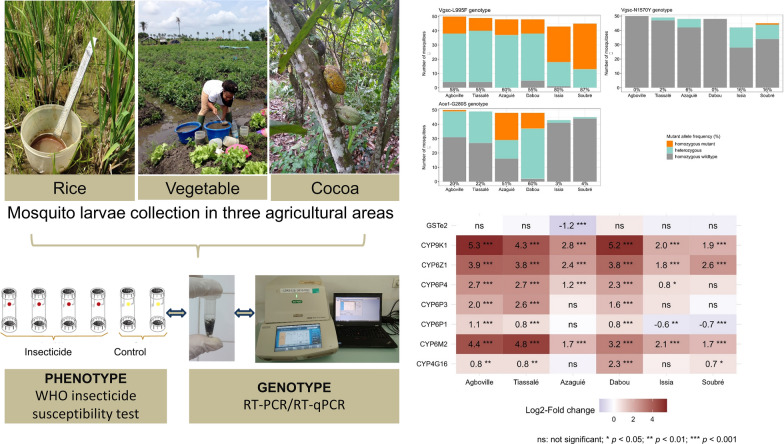

## Background

Vector control is the main strategy for controlling malaria and has shown success in Africa over many years. Unfortunately, vectors are becoming increasingly resistant to the insecticides used in public health. This threatens the efficacy of long-lasting insecticidal nets (LLINs) and indoor residual spraying (IRS), which are the main tools for controlling malaria mosquitoes [1]. The classical active compounds used for these tools are pyrethroids, organochlorines, organophosphates and carbamates. However, other active ingredients have recently been repurposed, including neonicotinoids and pyrroles [2]. Resistance to commonly used insecticides has been reported in malaria vectors in several sub-Saharan African countries [3–9].

In addition to behavioural changes, different physiological mechanisms are involved in insecticide resistance. The most important ones described in African malaria vectors are target-site resistance leading to alterations of the insecticide target sites, preventing the binding of the insecticide [10]; metabolic resistance, characterised by changes in insect enzyme systems leading to rapid detoxification or sequestration of insecticides [11]; and cuticular resistance that reduces the amount of insecticide penetrating the insect [12].

Target-site mutations in the voltage-gated sodium channel (*Vgsc*) are associated to pyrethroid and dichlorodiphenyltrichloroethane (DDT) resistance and are also known as knockdown resistance (*kdr*) mutations [6, 13, 14]. In *Anopheles gambiae* sensu lato, *kdr* is predominantly conferred by two mutations at the same codon position *Vgsc*-L995F/S [15, 16]. An additional mutation, called ‘super *kdr *’ (*Vgsc*-N1570Y), co-occurs with the L995F allele and increases resistance further [17]. In West Africa and particularly in Côte d’Ivoire, *kdr* is among the most commonly reported resistance mechanisms, with the *Vgsc*-995F allele predominating [6, 18–20]. Another point mutation in the acetylcholinesterase gene that causes a glycine to serine substitution (*Ace1*-G280S) and confers resistance to carbamate and organophosphate [21] has also been found in *Culex pipiens quinquefasciatus* [22] and *An. gambiae* [23, 24] mosquitoes from Côte d’Ivoire. While the *kdr* and *Ace1* target-site mutations are involved in insecticide resistance, they alone do not explain the highly resistant phenotypes observed in mosquitoes [25].

The other major physiological mechanism of insecticide resistance in mosquitoes is the overexpression of metabolic enzymes that detoxify or sequester insecticides [11]. Metabolic resistance includes, among others, enzymes from three major families of enzymes: the cytochrome P450-dependent monooxygenases (P450s or *CYPs*), carboxylesterase and glutathione *S*-transferases (*GSTs*). Overexpression of several detoxification genes related to insecticide resistance has been detected in field mosquito populations in Côte d’Ivoire, mostly from rice-cultivating areas [14, 26–28]. In 2014, Edi et al. [27] showed that some genes belonging to the *CYP6* P450 family metabolise pyrethroids and other insecticides in field mosquitoes from Tiassalé in southern Côte d’Ivoire. The involvement of P450s in the resistance of malaria vectors from the same locality has been repeatedly demonstrated in subsequent studies using synergist bioassays [25], quantitative polymerase chain reaction (qPCR) and RNA sequencing [14].

Although insecticide resistance in malaria vectors is attributed to the use of insecticides in public health, an increasing number of studies suggest that the use of pesticides in agriculture also contributes to the selection of insecticide resistance in mosquitoes [1, 18, 29, 30]. Growing human populations, particularly in Africa, exert increasing pressure on agricultural productivity, leading to intensified use of pesticides [31]. Indeed, importations of pesticides between 2005 and 2015 nearly tripled in the West African region, particularly in the three largest pesticide markets of Côte d'Ivoire, Ghana and Nigeria [32]. Furthermore, the uncontrolled and improper use of agrochemicals can lead to the development of insecticide resistance in non-target insects, including malaria vectors breeding in agricultural areas, since the compounds used to control crop pests often have the same active ingredients and molecular targets as those used in public health. [5]. Therefore, farming irrigation greatly increases the risk of malaria for nearby communities as well as the development of insecticide resistance by pesticides intended to control crop pests [33–35]. Cross-resistance to public health insecticides in mosquitoes is now a real obstacle for the current vector control methods adopted by the World Health Organization (WHO) Global Plan for Insecticide Resistance Management (GPIRM) [36]. These methods are primarily based on IRS and LLINs, and require a better understanding of the association between agricultural practices and insecticide resistance.

Previous studies have reported widespread use of pesticides in different types of agriculture and have shown that vector resistance to insecticides varies from one site to another in Côte d’Ivoire [18]. Although both target-site resistance and upregulation of P450 genes have been described in *An. gambiae* s.l. in the country, the association between different agricultural practices and insecticide resistance has not been studied. Therefore, assessing the current status of malaria vector resistance from different agricultural practices becomes a necessity to facilitate more effective planning of control strategies based on the type of agriculture. Here, we assessed the phenotypic resistance to discriminating concentrations of insecticides using the standard WHO insecticide susceptibility test in *An. gambiae* s.l. collected from rice, vegetable and cocoa cultivation areas in western Côte d'Ivoire. We further measured the frequency of target-site resistance alleles and characterised the expression levels of metabolic genes previously associated with insecticide resistance in the country.

## Methods

### Study sites

**Fig. 1:**
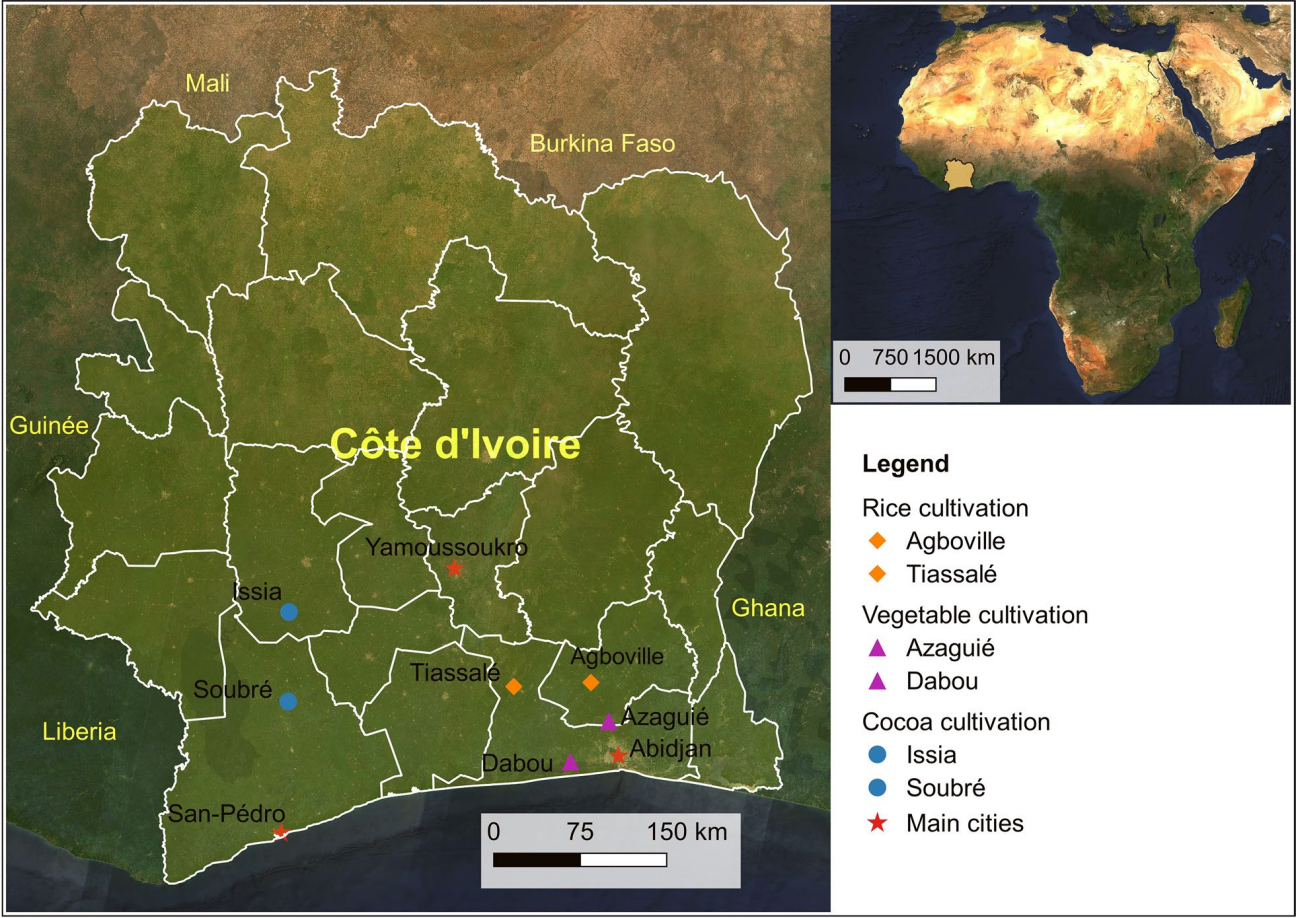
Map of the mosquito larval collection sites in Côte d’Ivoire. In Agbovile and Tiassalé, larvae were collected in the rice fields, while they were collected in the vegetable fields in Azaguié and Dabou. In the western part, mosquito larvae were collected in the cocoa fields of Issia and Soubré. The map was created with QGIS (2022, QGIS Geographic Information System. QGIS.ORG Association; http://www.qgis.org). Basemap source: Sentinel-2 cloudless (https://s2maps.eu) by EOX IT Services GmbH (contains modified Copernicus Sentinel data 2020)

Larval collections were carried out in six study sites in Côte d’Ivoire with three different agricultural practices, including cocoa, rice and vegetable cultivation. Each type of cultivation included two sites in the study. The sites with irrigated rice cultivation were in Agboville (latitude: 5.935496°, longitude: −4.223084°) and Tiassalé (latitude: 5.904263°, longitude: −4.826142°) located in the forest zones of Côte d’Ivoire. The vegetable sites were Azaguié (latitude: 5.633333°, longitude: −4.083333°) and Dabou (latitude: 5.316667°, longitude −4.383333°), also located in the forest zone, while the cocoa sites were Issia (latitude: 6.487614°, longitude: −6.583677°) and Soubré (latitude: 5.786623°, longitude: −6.589017°), which are characterised by evergreen forests (Fig. 1).

The climate in the study areas is humid and equatorial [37], characterised by four seasons: (i) a long rainy season that brings heavy rains from May to June; (ii) a short rainy season with rains from August to September in the southern part and from August to October in the more western part; (iii) a short dry season from October to November; and (iv) a main dry season from December to April. The average temperature varies between 21 °C and 33 °C.

To control dipteran and lepidopteran insects damaging the crops, mainly pyrethroids and neonicotinoids are deployed in the rice fields of Agboville and Tiassalé and the vegetable fields of Azaguié and Dabou [29]. Both agricultural practices are carried out throughout the year in Côte d’Ivoire. Issia and Soubré are located in the largest cocoa production region of Côte d’Ivoire. Cocoa production is the main economic activity in the western part of the country and one of the major pillars of Côte d’Ivoire’s economy. In cocoa-growing areas, neonicotinoids combined with pyrethroids are the insecticides predominantly used to protect cocoa fields against mirid infestations that are considered to be the most important pest problem in cocoa cultivation [18, 38]. In our study sites, the LLINs distributed in the irrigated rice and vegetable areas contain deltamethrin, while those distributed in the cocoa area contain alpha-cypermethrin [39].

### Larval collections and insecticide susceptibility bioassay

We collected *An. gambiae* s.l. larvae in southern Côte d’Ivoire (i.e. Agboville, Azaguié, Dabou and Tiassalé) from June to July in 2018 and in the western Côte d’Ivoire (i.e. Issia, Soubré) in June 2019. In Agboville and Tiassalé, we collected larvae in irrigated rice fields, while in Dabou and Azaguié, they were collected in water pits in the vegetable fields. In Soubré and Issia, we made collections in water puddles in cocoa fields (Fig. 1). Once back to Abidjan, we reared the larvae to the adult stage in the insectary of the Centre Suisse de Recherches Scientifiques en Côte d’Ivoire (CSRS) under standard conditions of 26 ± 2 °C, 75 ± 10% relative humidity and a 12 h/12 h light/dark photoperiod. Larvae from each site were reared separately and fed each morning with 0.075 g of TetraMin^®^ fish food powder (Tetra, Melle, Germany). Emerging adults had access to a 10% honey solution.

To determine the phenotypic insecticide susceptibility to the four classic insecticide classes commonly used in public health, we performed the standard WHO insecticide susceptibility test [40] with 2- to 5-day-old, non-blood-fed adult female mosquitoes that had emerged from the field-collected larvae. We tested their susceptibility against the WHO discriminating concentrations of bendiocarb (0.1%), deltamethrin (0.05%), DDT (4%) and malathion (5%) on treated filter papers sourced from WHO. To determine the knockdown ratio, we conducted bioassays with adults taken from an insecticide susceptible *An. gambiae* sensu stricto. Kisumu colony and exposed them to deltamethrin (0.05%) and DDT (4%). For each combination of insecticide and field site, we exposed six batches of 20–25 females including four batches exposed to insecticide-impregnated filter papers and two batches serving as negative controls and exposed to control papers containing only the insecticide carrier oil. During the 1 h exposure time, we recorded how many mosquitoes were knocked down at 5-min intervals. After an hour of exposure, we transferred the mosquitoes back to the holding tubes and allowed them to feed on 10% honey solution ad libitum, while the delayed mortality was recorded 24 h post-exposure.

### Preparation of samples and extraction of nucleic acids for molecular analysis

We killed the mosquitoes that were still alive 24 h post-exposure in absolute ethanol and then blotted away any excess ethanol with a paper towel. We then gently transferred the mosquitoes by batches of 10 to 100 individuals into 1.5 ml microcentrifuge tubes that contained 0.7–1.4 ml of RNA*later*^®^ (Ambion, Inc., Austin, TX, USA) depending on the number of mosquitoes. We kept RNA*later* tubes with mosquitoes overnight at 4 °C to allow for thorough penetration of the tissue. The following day, the excess RNA*later*^®^ was removed and the tubes were stored at −20 °C until further processing for DNA and RNA extraction.

For the extraction of total RNA and DNA, we randomly picked 50 RNA*later*^®^-preserved individuals from the controls that had not been exposed to insecticides in the bioassays and processed them using the MagnaMedics magnetic bead-based kit (MagnaMedics GmbH, Aachen, Germany). We ground the mosquitoes individually in 1.5-ml microcentrifuge tubes by adding 200 µl TE buffer (10 mM tris(hydroxymethyl) aminomethane hydrochloride [Tris–HCl], 1 mM ethylenediaminetetraacetic acid [EDTA], pH 8.0) per tube and using a pestle, driven by a hand-held tissue grinder. Then, we added 150 µl lysis buffer, mixed everything by vortexing for 15 s and incubated the mixture for 10 min at room temperature while vortexing the tubes every 2 min for 15 s. Following the incubation, we spun down the non-lysed mosquito debris by centrifugation at 16,000×*g* for 2 min. Next, we transferred the clear supernatant into a new 1.5-ml tube, added 20 µl magnetic beads and 440 µl binding buffer, and vortexed the mixture for 15 s. We incubated the mixture again for 10 min at room temperature while vortexing the tubes in between as described above for the lysis step. The tubes were placed on a magnetic rack for 2 min, allowing for the magnetic beads to sediment, and then discarded the supernatant. We washed the remaining beads twice by adding 200 µl wash buffer, vortexed the tubes and let the mixture incubate for 1 min at room temperature before placing it for 2 min back on the magnetic rack and discarding the supernatant. Finally, we extracted the nucleic acids by adding 180 µl elution buffer, vortexed the tubes and incubated the mixture for 10 min in a water bath set at 50 °C and vortexed in between as above. After the tubes were removed from the water bath, we vortexed them again, spun them down and placed the tubes on the magnetic rack for 2 min. Finally, we collected the supernatant that now contained the purified DNA and RNA, transferred the solution to new 1.5-ml tubes and stored them at −80 °C.

### Molecular analysis

To identify the sibling species of the *An. gambiae* s.l. complex, we performed two TaqMan^®^ multiplex qPCR assays [41, 42] with modifications to the original protocols as described in Wipf et al. [14]. In brief, the first assay differentiates *Anopheles coluzzii* and *An. gambiae* s.s. as a group (Ag+) from *Anopheles bwambae*, *Anopheles melas*, *Anopheles merus* and *Anopheles quadriannulatus* (Aq+) and from *Anopheles arabiensis* (Aa+). The second assay distinguishes between *An. coluzzii* (former molecular M-form) and *An. gambiae* s.s. (former molecular S-form) based on the SINE 200 X6.1 locus that is fixed in *An. coluzzii* and absent in *An. gambiae* s.s. We used the common primers designed by Santolamazza et al. [42] and the probes described in Wipf et al. [14]. We ran the second assay on the DNA from the samples that turned out to be Ag+ in the first assay.

In addition to the species identification assays, we applied diagnostic TaqMan^®^ qPCR assays to detect the target-site *kdr* mutations of the voltage-gated sodium channel (i.e. *Vgsc*-L995F/S and *Vgsc*-N1570Y) and the acetylcholinesterase mutation *Ace1*-G280S, following the protocol of Bass et al. [43] with the adaptations described in Mavridis et al. [44].

In addition to the diagnostic qPCRs for species identification and the detection of target-site mutations, we measured the expression levels of genes that have previously been associated with insecticide resistance in *An. gambiae* s.l., including the cytochrome P450-dependent monooxygenases *CYP4G16*, *CYP6M2*, *CYP6P1*, *CYP6P3*, *CYP6P4*, *CYP6Z1* and *CYP9K1*, and the glutathione *S*-transferase *GSTe2*. As a reference for the overall gene expression, we measured additionally the expression levels of the housekeeping gene encoding for the ribosomal protein S7 (*RPS7*). Again, we used a series of messenger RNA (mRNA)-specific TaqMan^®^ reverse transcription qPCRs (RT-qPCRs) that were developed by Mavridis et al. [45]. Instead of pooling RNA or DNA, we measured the gene expression levels separately for each individual with 50 individuals per field site and 50 individuals from the insecticide-susceptible *An. gambiae* s.s. Kisumu colony. This susceptible strain from Kenya is kept in the insectary as a control for insecticide susceptibility bioassays.

We ran the qPCR reactions in volumes of 10 µl, containing 1 µl of template nucleic acid extract and 9 µl of master mix comprising primers and probes at final concentrations as published previously [14]. The master mix reagents were supplied by Fast-Track Diagnostics (FTD, Esch-sur-Alzette, Luxembourg). All reactions were performed on a C1000 Touch/CFX96™ Real-Time PCR System (Bio-Rad Laboratories, Hercules, CA, USA) in 96-well plates (Sarstedt, Nümbrecht, Germany; catalogue number: 72.1980.202). The thermal cycle parameters were 15 min for the reverse transcription at 50 °C, RTase inactivation and initial denaturation at 95 °C for 3 min, followed by 40 cycles of denaturation at 95 °C for 3 s and annealing/extension steps at 60 °C for 30 s.

### Data analysis

For the insecticide susceptibility assay, we scored both the immediate knockdown at 5-min intervals up to 1 h and the delayed mortality rates at 24 h post-exposure. Survival analysis was performed to visualise pyrethroid and DDT knockdown time, comparing the knockdown effect on the field mosquito populations to the one on the insecticide susceptible Kisumu colony. For the interpretation of the mortality rates in terms of phenotypic resistance status, we followed the WHO criteria [40]: a mortality rate below 90% indicates resistance; a mortality rate equal to or above 98% indicates susceptibility; and a mortality rate between 90 and 97% is suggestive of possible resistance that needs to be confirmed.

The expression levels of the genes of interest relative to the internal control (RPS7) were calculated using the comparative threshold cycle (C_T_) method, also referred to as the 2^−ΔC^_T_ method, where ΔC_T_ = C_T gene of interest_—C_T internal control_ [46]. To assess whether gene expression levels differ between mosquito populations, we ran a linear regression model for each gene with 2^−ΔCT^ as the dependent variable. We used the susceptible Kisumu strain as the reference. We set the level of significance at *α* = 0.05 and adjusted the *P*-values for multiple testing using the Bonferroni adjustment method [47].

We ran all data analysis in R version 4.0.3 [48] using RStudio version 1.3.1093 [49]. We used the ‘tidyverse’ R package for data tidying and visualisation [50]. The R packages ‘survival’ and ‘survminer’ [51] were used to plot the survival curves and to calculate the Kaplan–Meier estimates [52].

## Results

### Insecticide susceptibility assays

**Fig. 2:**
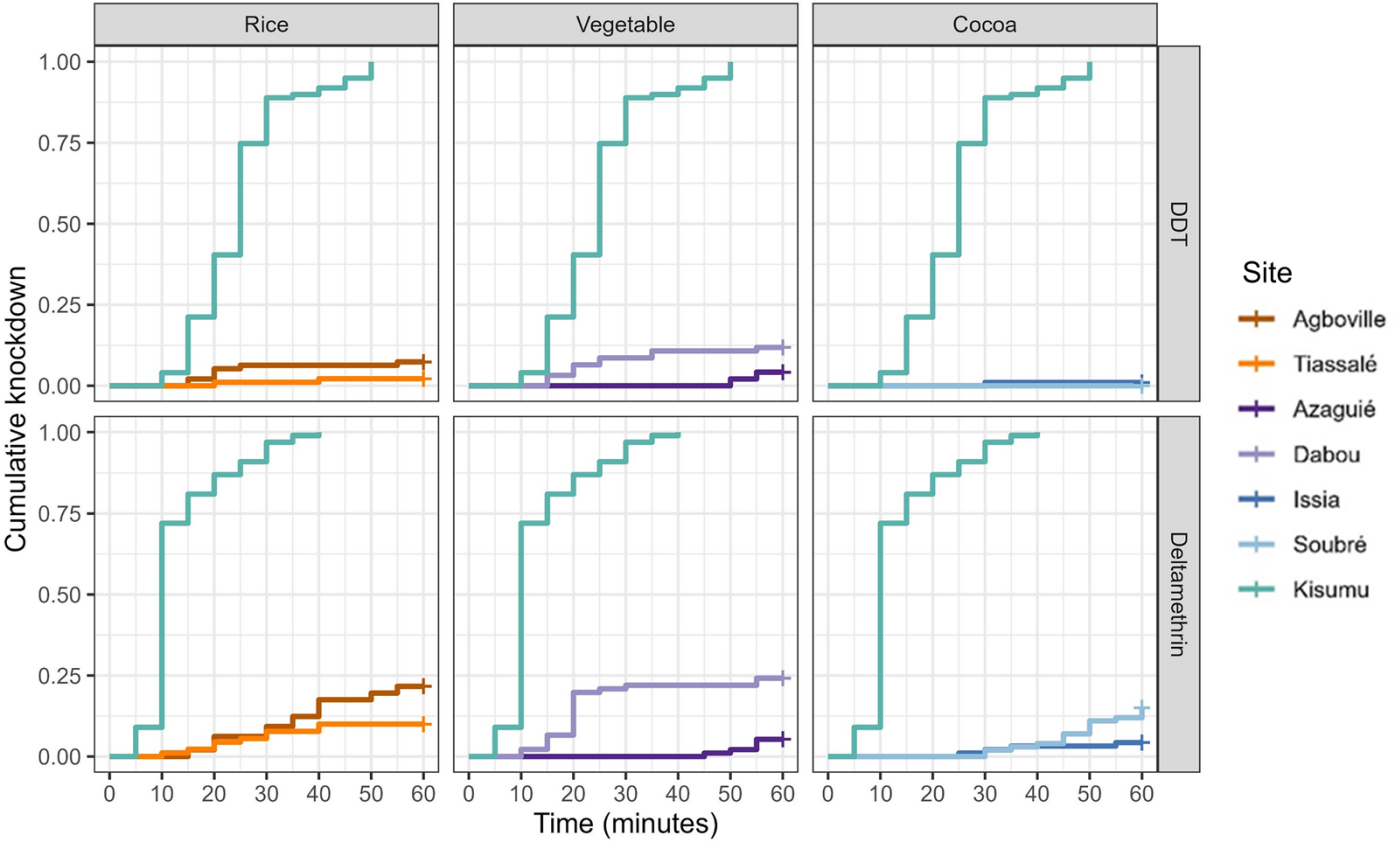
Kaplan–Meier ‘survival’ curves showing the cumulative knockdown over the 60 min exposure to diagnostic concentrations of deltamethrin (0.05%) and DDT (4%) in the WHO insecticide susceptibility assay. The log-rank test used for Kaplan–Meier test showed *P*-values < 0.0001 between Kisumu and rice, vegetable and cocoa sites, respectively

**Fig. 3:**
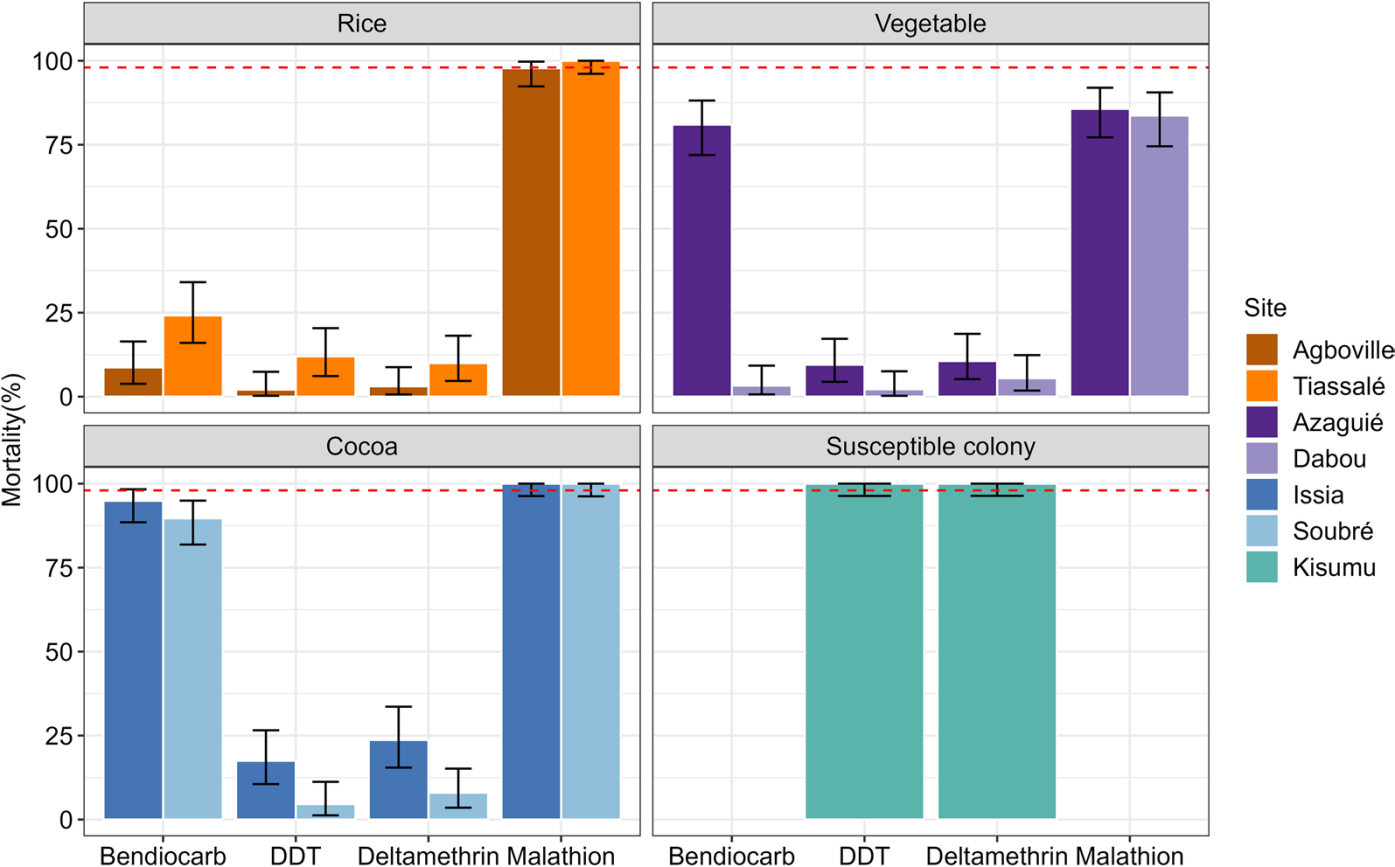
24 h mortality rates against bendiocarb (0.1%), DDT (4%), deltamethrin (0.05%) and malathion (5%) diagnostic concentrations. Mortality rates above 98% (red line) indicate susceptibility according to WHO criteria of phenotypic resistance. Error bars indicate 95% confidence intervals

The knockdown rates for DDT and deltamethrin varied significantly between the sites (DDT: *P*-value < 0.001; deltamethrin: *P*-value < 0.0001) but were substantially lower in all field populations when compared to the insecticide-susceptible Kisumu colony (*P*-value < 0.0001) (Fig. 2) confirming the insecticide resistance observed in the delayed 24 h mortality rates (Fig. 3). Deltamethrin exposure knocked down mosquitoes from all tested field and lab populations more rapidly than DDT (Fig. 2). Among the field populations, the population from the Dabou vegetable site showed the fastest and highest knockdown rate with both deltamethrin and DDT although endpoint knockdown rates 1 h post-exposure did not exceed 25% and 12%, respectively. The lowest knockdown rate with deltamethrin was observed in the Issia cocoa field population. In contrast, the majority of the Kisumu mosquitoes were already knocked down after 10 min exposure to deltamethrin and all of them after 40 min (Fig. 2). The knockdown probability with DDT was the lowest (0%) in Soubré cocoa field populations and highest (12%) in Dabou vegetable field populations (*P*-value < 0.0001) (Fig. 2). In comparison, half of the susceptible Kisumu mosquitoes were knocked down by DDT after 25 min and all after 50 min exposure time (Fig. 2).

All mosquito populations from the three agricultural practices were resistant to deltamethrin, DDT and bendiocarb. However, the populations from the rice and cocoa sites were still susceptible to malathion (Fig. 3). The Kisumu reference colony was fully susceptible to DDT and deltamethrin. The 24 h mortality rates in the non-exposed control groups were below 5% in each bioassay and thus we did not correct for control mortalities.

### Molecular analysis

Across all sites, the qPCR assays confirmed the presence of both *An. gambiae* s.s. and *An. coluzzii* with a 96% predominance of *An. coluzzii* (*n* = 295). All mosquitoes from the rice fields of Agboville and Tiassalé as well as those from the vegetable farms of Azaguié and Dabou were identified as *An. coluzzii,* except for one *An. coluzzii*/*An. gambiae* s.s. hybrid from Azaguié. While *An. coluzzii* was the predominant species in the cocoa plantations of Issia and Soubré, we identified 12% and 10% of the specimens as *An. gambiae* s.s., respectively.

**Fig. 4:**
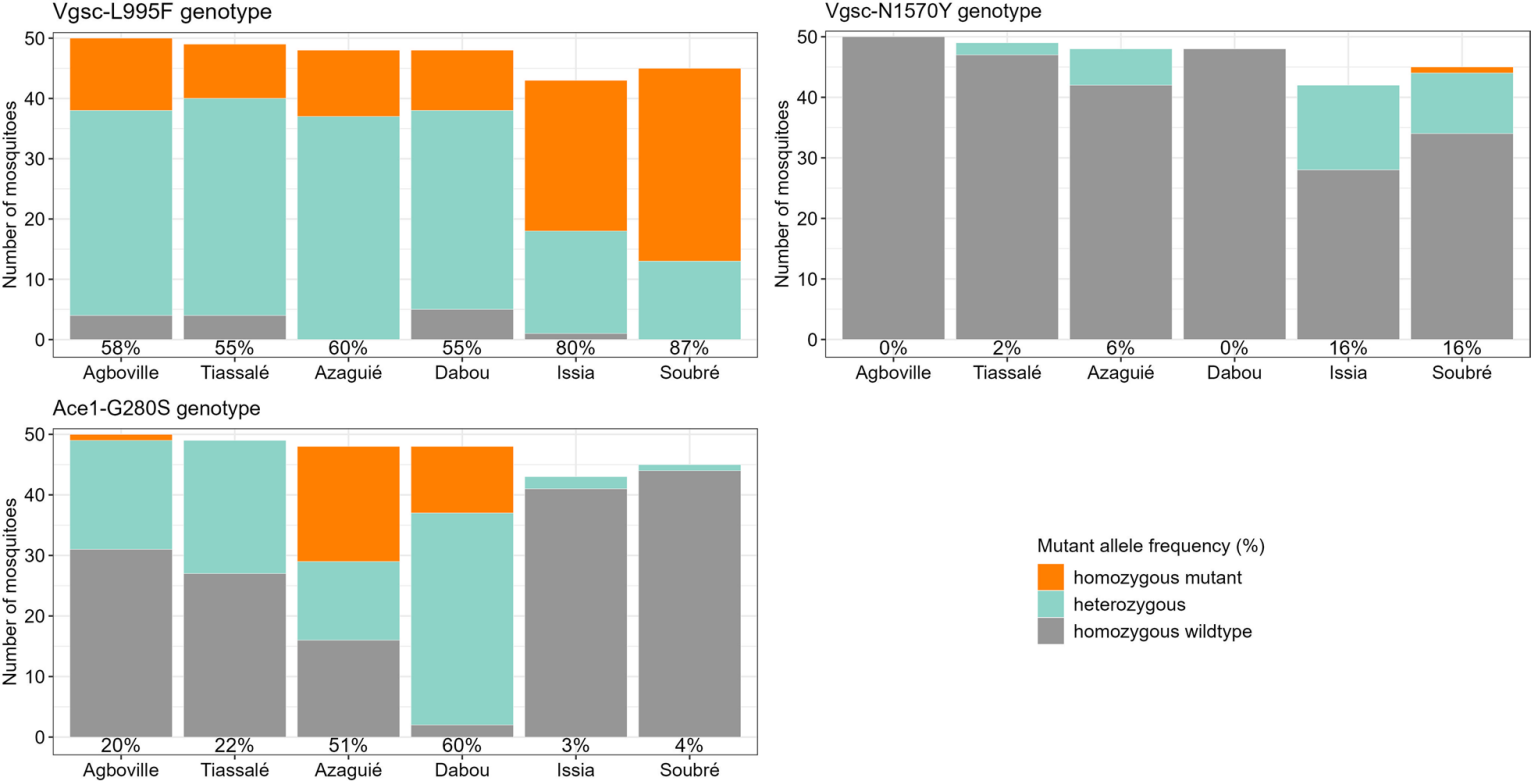
Allelic frequency of target-site resistance mutations *Vgsc*-L995F, *Vgsc*-N1570Y and *Ace1*-G280S. The percentages indicate the frequency of the resistance alleles while the bars show the actual numbers

We identified several target-site mutations, including the *kdr* loci *Vgsc*-L995F and *Vgsc*-N1570Y and the acetylcholinesterase mutation *Ace1*-G280S, while the allelic frequency varied by site and agricultural practice (Fig. 4). In contrast, we did not detect the mutant *Vgsc*-L995S allele. The mutation *Vgsc*-L995F that is associated with pyrethroid and DDT resistance was the most frequent allele across all sites (Fig. 4). We detected high *Vgsc*-L995F allelic frequencies in the cocoa-growing sites of Issia and Soubré, whereas we found moderate frequencies in the vegetable and rice sites. The *Vgsc*-N1570Y mutation was present at low frequencies in Azaguié, Issia, Soubré and Tiassalé and undetected in Agboville and Dabou (Fig. 4). The highest frequencies were found in both cocoa-growing sites.

We found the *Ace1*-G280S mutation associated with carbamate and organophosphate resistance at the highest allelic frequencies in both the Dabou (60%) and Azaguié (51%) vegetable-growing sites but mostly in Dabou, while it was under 25% in the rice field populations of Agboville and Tiassalé, and under 5% in the cocoa fields of Issia and Soubré (Fig. 4).

**Fig. 5:**
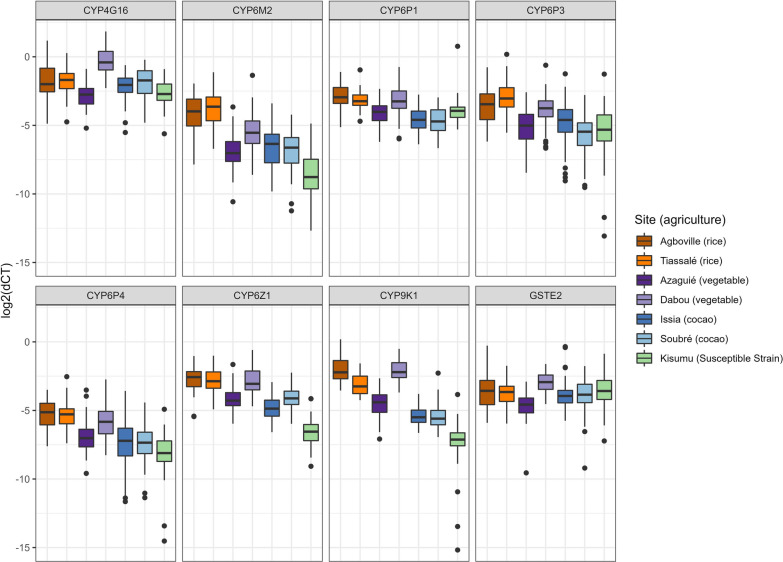
Gene expression levels of detoxification enzymes in *Anopheles gambiae* s.l. from the six field populations with three different agricultural practices and in the Kisumu susceptible strain. The boxes indicate the 25–75% quartiles. The whiskers show the 5–95% range and the dots represent outliers

**Fig. 6:**
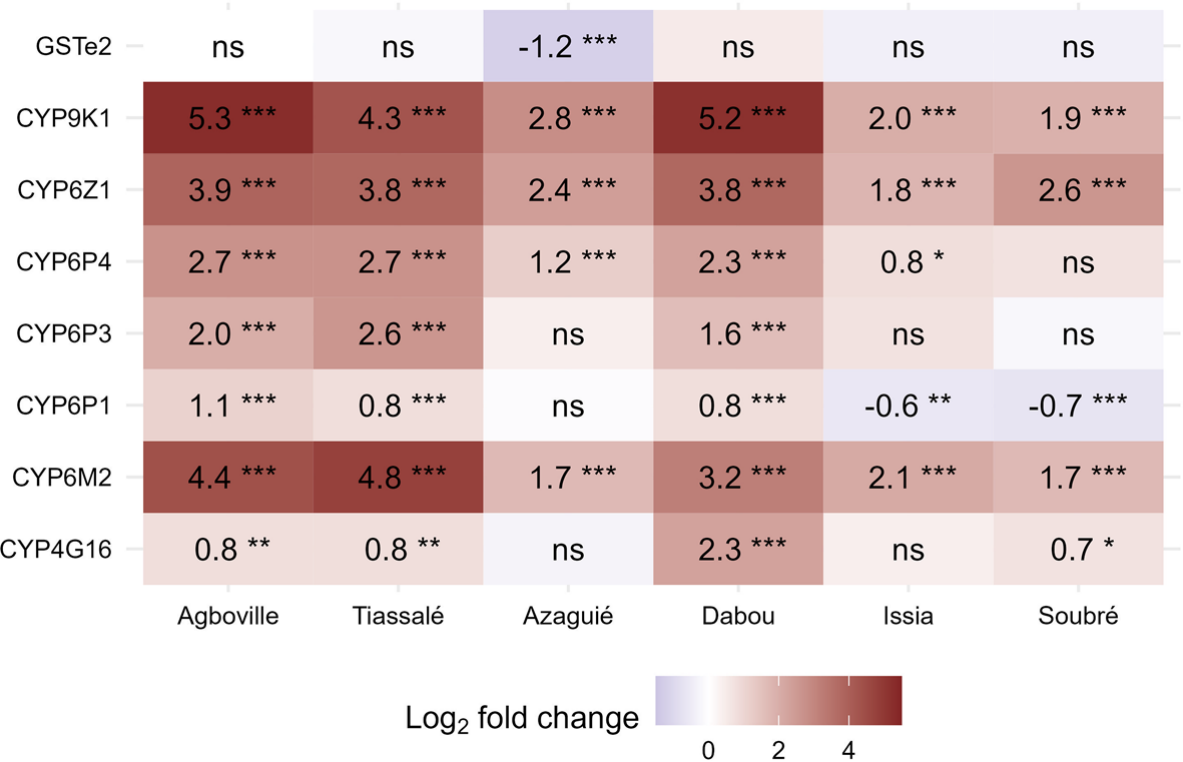
Differential expression levels for measured putative detoxifying genes across field populations. The fold changes give the change in expression level of a population against the susceptible reference strain Kisumu on the log_2_ scale. The fold changes were estimated using generalised linear regression models for each gene. *ns* not significant; * *P* < 0.05; ** *P* < 0.01; *** *P* < 0.001

The cytochrome P450-dependent monooxygenases *CYP4G16*, *CYP6M2*, *CYP6P1*, *CYP6P3*, *CYP6P4, CYP6Z1* and *CYP9K1* were significantly overexpressed compared to Kisumu with variations across the different agricultural sites (Figs. 5 and 6). Cocoa-growing sites showed overall low expression levels, while rice-growing sites had increased expression levels and vegetable-growing sites showed a mixed picture (Fig. 6). The three P450s *CYP9K1*, *CYP6Z1 and CYP6M2* were significantly overexpressed in all six field sites while being most notably overexpressed in the rice-growing sites of Agboville and Tiassalé followed by the vegetable site of Dabou (Fig. 6). The highest fold changes were detected for *CYP9K1* in Agboville (5.3-fold up) and Dabou (5.2-fold up). Interestingly, while *CYP4G16* was upregulated in Dabou, the same gene was not significantly differentially expressed in the other vegetable-growing site Azaguié and the cocoa-growing site Issia.

## Discussion

Regardless of the prevailing agricultural practice, *An. gambiae* s.l. populations from all six Ivorian sites were resistant to deltamethrin, DDT and bendiocarb, whereas only populations from vegetable-growing areas were additionally resistant to malathion. While *kdr* mutations altering the pyrethroid and organochlorine target site appeared as the driving insecticide resistance mechanism in cocoa sites, we identified high expression levels of insecticide-metabolising P450 enzymes in the irrigated rice sites. In the two vegetable sites, we found both *kdr* and *Ace1* target-site mutations associated with phenotypic resistance but the sites showed contrasting expression patterns of metabolic genes.

In Côte d’Ivoire—as in other African countries—it has been suggested that the use of pesticides in agriculture is one of the causes indirectly related to insecticide resistance in malaria vectors, although few studies have focused on the relationship between agriculture and insecticide resistance [29, 30, 53, 54]. Most of the previous studies conducted in Côte d’Ivoire have shown a very high trend of resistance to pyrethroids and DDT and also resistance to carbamates and organophosphates is progressively increasing [27, 28, 55]. It was found in a monitoring study that pyrethroids are predominant among the insecticides used in vegetable, rice and cocoa fields while vectors from these agricultural practices showed resistance to DDT, deltamethrin and bendiocarb [18]. In 2016, Chouaïbou et al. [29] detected malathion resistance in malaria vectors collected from vegetable fields in Dabou and found that organophosphates accounted for 9% of insecticides used in this site compared to only 2% in the rice fields of Tiassalé, where mosquitoes were susceptible to malathion, like in our study. Thus, agricultural pesticides in mosquito breeding habitats may favour the selection of insecticide resistance in *An. gambiae* s.l. mosquitoes against compounds that were not used in vector control interventions.

In the search for the mechanisms causing the observed phenotypic resistance, we found a very high allelic frequency of the *Vgsc*-L995F mutation associated to pyrethroid and DDT resistance in cocoa areas. Indeed, Côte d’Ivoire being the first world cocoa producer, its western part is in the process of becoming the new zone of high cocoa production of the country [37]. Thus, pesticides such as pyrethroids are intensively applied in cocoa farming to protect them against crop pests [38]. These activities and the deployment of alpha-cypermethrin-treated nets during interventions of the national malaria control programme in this region [39] likely exerted enormous selection pressure, leading to the development of pyrethroid resistance in the vectors. Moreover, we detected at moderate frequency the presence of the *Vgsc*-1570Y allele that amplifies the effects of the *Vgsc*-L995F mutation. To our knowledge, this is the first time that the *Vgsc*-N1570Y mutation has been detected in Issia and Soubré cocoa fields. The findings of this study show that the phenotypic resistance observed in the cocoa region is probably associated with the high frequency of the *Vgsc*-L995F and *Vgsc*-N1570Y mutation, likely confirming the crucial role of the *kdr* mutation in conferring pyrethroid and DDT resistance.

We observed resistance to carbamate and organophosphate insecticides in *An. gambiae* s.l. populations from vegetable fields of Dabou and Azaguié. This may be indicative of intensive use of carbamate and organophosphate, which can pollute mosquito breeding sites in the cultivated environment. This hypothesis is supported by the study of Chouaïbou et al. [29] that found carbamate and organophosphate residues in mosquito breeding sites and in the soil in Dabou vegetable fields as well as phenotypic resistance in *An. gambiae* s.l. to bendiocarb and malathion. Reassuringly, we found the highest frequencies of the insensitive acetylcholinesterase *Ace1*-G280S gene in mosquitoes from vegetable areas, confirming the cross-resistance observed between carbamate and organophosphate insecticides. In 2016, Chouaïbou et al. [29] detected malathion resistance in malaria vectors collected from vegetable fields in Dabou and found that organophosphates accounted for 9% of insecticides used in this site compared to only 2% in the rice fields of Tiassalé, where mosquitoes were susceptible to malathion as in our study. Therefore, the presence of *Ace1* mutation conferring cross-resistance to these insecticides represents an important threat for carbamate and organophosphate-based vector control strategies. The use of newly developed insecticides with different modes of action and their combination with older classes in large-scale interventions is urgently needed to manage insecticide resistance.

Finally, gene expression analysis revealed that several P450s were overexpressed in rice fields relative to Kisumu. We found that P450s *CYP9K1*, *CYP6M2*, *CYP6Z1*, *CYP6P4* and *CYP6P3* were the most upregulated among the genes detected in rice-growing sites and vegetable-growing sites. In fact, frequent exposure of larvae to agricultural pollutants and several xenobiotics in the water of rice and vegetable fields could induce metabolic stress resulting in insecticide detoxification in the insect. Recent studies have shown that recurrent exposure of *An. gambiae* s.l. and *Aedes aegypti* larvae to agrochemicals induced significant tolerance to insecticides due to a stimulation of multiple genes responsible for target-site resistance and metabolic resistance, including P450 genes [56, 57]. Thus, the practice of rice growing and vegetable growing several times a year and the intensive use of insecticides in these crops, as well as the xenobiotics present in the crop water pits favouring metabolic gene expression, may be why P450 mechanisms are more strongly selected in rice-growing sites and in the vegetable-growing site of Dabou than the cocoa-growing sites. The overexpression of several P450s revealed in both the rice-growing sites and vegetable-growing site of Dabou, alongside the target-site mutations, may explain the low DDT, deltamethrin and bendiocarb mortalities observed. We found that *CYP6M2* was upregulated in all sites, but the gene was mostly overexpressed in Tiassalé compared to Kisumu. In fact, Tiassalé field mosquitoes were found resistant to deltamethrin, whereas they were still susceptible to malathion despite the presence of *Ace1* mutations, which might be an indication for negative cross-resistance as discussed in Wipf et al. [14]. The evidence that P450 enzymes (*CYP6M2*) can confer negative cross-resistance has also been provided directly in an in vivo study with African malaria vectors by Adolfi et al. [58]. Such increased susceptibility to malathion may have a positive impact on insecticide resistance management—especially for the improvement of malaria vector control tools [59].

Although we observed some associations between insecticide resistance mechanisms and agricultural practice, we acknowledge that several other factors may have influenced the studied outcome. A major caveat is that the majority of our field sites with the same agricultural type were geographically closer to each other than to those of another type. Additionally, the larval collections in the cocoa-growing areas were done a year later than in the rice- and vegetable-growing areas. Both limitations above may have led to similar environmental factors—in addition to those stemming from agriculture—influencing resistance selection in malaria vectors. For example, the LLINs distributed in the different geographical areas were treated with different pyrethroid insecticides [60]. Future studies would greatly benefit from measuring the strength of resistance to different insecticides using intensity or dose–response bioassays and including the novel insecticides prequalified by WHO: broflanilide, chlorfenapyr and clothianidin [61].

Although recent studies have shown overexpression of detoxification genes in some of our study sites but mostly in pooled mosquito samples, this study went a step further by measuring the gene expression levels of each individual separately, strengthening our results and providing entomological baseline data for rice-, vegetable- and cocoa-growing areas.

The insecticide resistance observed in all cultivation areas would constitute an obstruction to the various control strategies that are essentially based on LLINs and IRS if nothing is done to reverse the current situation. The involvement of other environmental factors such as particular pollutants and waste from industries established around crop areas needs to be thoroughly investigated to determine whether these factors contribute to the selection pressure of the metabolic and target site resistance mechanism in malaria vectors around farms. In conclusion, the present study revealed intriguing associations between agricultural practice and the type of resistance mechanisms in malaria vectors that merit further exploration. Therefore, it is suggested that national malaria control programmes collaborate more closely with the agricultural sector to jointly develop integrated risk management and vector control strategies involving farmers.

## Data Availability

All relevant data are included within the paper. Raw data are available from the corresponding author upon reasonable request.

## Abbreviations

*Ace1*: Acetylcholinesterase
*An. gambiae*: *Anopheles gambiae*
*An. gambiae * s.l.: *Anopheles gambiae* sensu lato
*An. gambiae* s.s.: *Anopheles gambiae* sensu stricto
CSRS: Centre Suisse de Recherches Scientifiques en Côte d’Ivoire
C_T_: Threshold cycle
DDT: Dichlorodiphenyltrichloroethane
EDTA: Ethylenediaminetetraacetic acid
GPIRM: Global Plan for Insecticide Resistance Management
GST: Glutathione *S*-transferase
IRS: Indoor residual spraying
*Kdr*: Knockdown resistance
LLINs: Long-lasting insecticidal nets
P450s: Cytochrome P450-dependent monooxygenases
RT-qPCR: Reverse-transcription quantitative (or real-time) polymerase chain reaction
Tris–HCl: Tris(hydroxymethyl) aminomethane hydrochloride
*Vgsc*: Voltage-gated sodium channel
WHO: World Health Organization
